# Regulatory-required post-marketing database studies in Japan could be leveraged to assess important potential risks as well as identified risks

**DOI:** 10.3389/fphar.2025.1565314

**Published:** 2025-07-21

**Authors:** Hiroshi Yamazaki, Ko Nakajo, Naoki Hirose, Li Yan, Sun Yeop Lee, Yongjing Zhang, Hong Qiu, Chieko Ishiguro

**Affiliations:** ^1^ Global Epidemiology, Johnson & Johnson, Tokyo, Japan; ^2^ Global Epidemiology, Johnson & Johnson, Beijing, China; ^3^ Global Epidemiology, Johnson & Johnson, Seoul, Republic of Korea; ^4^ Global Epidemiology, Johnson & Johnson, Titusville, NJ, United States; ^5^ Laboratory of Epidemiology Section, Department of Data Science, Center for Clinical Sciences, Japan Institute for Health Security, Tokyo, Japan

**Keywords:** post-marketing surveillance, drug safety, database study, Japan, pharmacovigilace, risk management plan

## Abstract

**Background:**

To investigate the characteristics of post-marketing database studies (PMDS) included in risk management plans (RMPs) across all therapeutic areas in Japan.

**Methods:**

Two researchers systematically and independently reviewed all RMPs listed on the Pharmaceuticals and Medical Devices Agency website from April 2013 to December 2023. PMDS contained in RMPs were identified, reviewed, and summarized by study design, target objectives, and data source. Specific objectives were linked to the data source.

**Results:**

Among 648 RMPs retrieved/reviewed, 85 PMDS were identified from 63 RMPs targeting 138 safety and five effectiveness objectives. Among 85 PMDS, 57 (67.1%) PMDS targeted important identified risk and 29 (34.1%) targeted important potential risk. Cohort studies were the most prevalent study design (74/85, 87.1%), and 74.1% (63/85) included a comparator group. Common target safety objectives included “infections and infestations”, “metabolism and nutrition disorders”, “cardiac disorders” and “vascular disorders”. The Medical Data Vision database was the most frequently used data source for PMDS (32/85, 37.5%) followed by the Medical Information Database Network (MID-NET^®^) (18/85, 21.2%) and JMDC (9/85, 10.6%)

**Conclusion:**

In Japan, PMDS are usually cohort studies with targeted safety objectives. Most studies currently target important identified risk rather than important potential risk and may not make full use of the advantages of PMDS that can include large populations, comparator groups, and that can assess the occurrence of rare adverse events. These results could be informative for pharmaceutical companies planning post-marketing studies as pharmacovigilance activities. Early public availability of PMDS protocols would promote improved study methodology and could potentially improve the scientific value of PMDS in Japan.

## 1 Introduction

Post-marketing studies are mostly conducted to investigate specific questions about safety and/or effectiveness of medicines and vaccines when administered to large numbers of individuals in real-world settings. These studies are variously described as post-marketing surveillance (PMS) studies in Japan, post-authorization studies in the European Union (EU), and post-marketing commitments and post-marketing requirements in the United States (European Medicines Agency, 2009; European Medicines Agency, 2012; Japan Pharmaceutical Manufacturers Association, 2020; US Food and Drug Administration, 2024). A revision of the Japan’s Pharmaceutical Affairs Law in 1980 set out requirements for PMS for nearly all new medicines or new indications with the aim of describing the incidence of common adverse drug reactions in the Japanese population. As a result, single arm, prospective observational PMS with primary data collection have been conducted in Japan for decades (Japan Pharmaceutical Manufacturers Association, 2009).

In 2005, the International Conference on Harmonization E2E guideline described the potential contribution of real-world data (RWD), such as that collected by registries, electronic medical records, and administrative claims databases, to pharmacovigilance, and over the last two decades, the use of RWD to support post-marketing activities has increased rapidly (ICH, 2004; Mofid et al., 2022). For example, in the EU, nearly three-quarters of post-authorization studies adopting an observational study design utilized RWD during the period from 2010 to 2018 (Sultana et al., 2022).

In Japan, limited access to data sources and limited real-world evidence capabilities in past years meant that RWD was infrequently considered as an option for PMS. More recently, the use of RWD to support PMS studies has been actively promoted by the Pharmaceuticals and Medical Devices Agency (PMDA) (Ishiguro and Uyama, 2019). In 2014, the PMDA published the first guidance on the use of RWD for pharmaco-epidemiological studies to assess aspects of safety relating to medicines (Pharmaceuticals and Medical Devices Agency, 2014). Subsequently, Good Post-marketing Study Practice (GPSP) guidance enforced in Japan for conducting of PMS studies was amended in 2018 to allow RWD to be leveraged in PMS studies conducted as part of pharmacovigilance activities in the Risk Management Plan (RMP) (Ministry of Health Labour and Welfare, 2017a; b). At the same time, the PMDA also made the Medical Information Database Network (MID-NET^®^) (Yamaguchi et al., 2019), which contains electronic medical records and administrative claims data from advanced hospitals, available to pharmaceutical companies. In addition to the MID-NET^®^, Medical Data Vision (MDV) and the JMDC are maintained by commercial database vendors, and are accessible databases for pharmaceutical companies considering employing RWD in PMS studies (Nagai et al., 2021; Laurent et al., 2022). Databases used in PMS must meet quality standards stipulated in GPSP guidance (Ministry of Health Labour and Welfare, 2018). Thus, there are several accessible database options for pharmaceutical companies that can be selected according to study objectives and database characteristics.

A review by the PMDA in 2022 to assess the regulatory impact of the GPSP revision found that the proportion of database studies had increased, but there continued to be a high number of single cohort observational studies that relied on primary data collection (Kohama et al., 2023). This could be due to inadequate real-world evidence capabilities, limited access to RWD by pharmaceutical companies, or limitations in the available databases to address research questions (Ishiguro and Uyama, 2019). Studies requiring primary data collection may be burdensome for healthcare professionals (Maeda et al., 2015), require considerable investment by pharmaceutical companies in terms of personnel and costs, and may be of prolonged duration, particularly if the target outcome is rare (Hiroi et al., 2017; Kohama et al., 2023; Wolter et al., 2024). By contrast, for appropriate target research questions, post-marketing database studies (PMDS) which use routinely collected data or data from registries, can be a more efficient and cost-effective alternative. Potential advantages of PMDS include access to very large sample sizes, availability of longitudinal data, and earlier availability of results (Ministry of Health Labour and Welfare, 2017c).

No comprehensive review of the target objectives and the type of databases employed in PMDS in Japan currently exists. Such information could be used by pharmaceutical companies and the PMDA to guide study objectives, design, and data sources when planning PMDS as part of an RMP. In this study, we aimed to describe characteristics of PMDS including the target objectives and data source used in all PMDS identified in RMPs from April 2013 to December 2023.

## 2 Methods

### 2.1 Identification of PMDS and data extraction from RMPs and PMDA review reports

All RMPs from April 2013 until 21 December 2023 were retrieved from the PMDA website, and PMDS were identified independently by two researchers from those RMPs. For RMPs with at least one PMDS, we collected information that included product regulatory details from the PMDA review report (Pharmaceuticals and Medical Devices Agency, 2021). Discrepancies were resolved discussion between the researchers or with the wider team until consensus. Information about the product regulatory details, including name of active ingredient, name of marketing authorization holder, and approval date, and details of the PMDS, including the types of objectives, terms of objectives, study design, comparator group, and source database, were extracted from the RMP. We also extracted regulatory characteristics, such as the type of New Drug Application (NDA) and applicability to orphan drug/pediatric indications from the PMDA review report.

### 2.2 Assessment of medicines investigated in PMDS

NDAs were classified into three categories; a new molecular entity (defined as a new active ingredient and/or new combination drugs), a partial change, or a generic drug. PMDA review reports were used to identify orphan drug or pediatric drug status, and the re-examination period, a period of 4 years, 6 years, 8 years, 10 years or the period remaining for other indications, at which point information on safety and effectiveness is required to be re-submitted for further assessment. Generic drugs are not subject to re-examination. Marketing authorization holders were classified as Japanese or foreign companies. The drug class was categorized according to the Anatomical Therapeutic Chemical Classification System code.

Pharmacovigilance activities listed in the RMP that were additional to the PMDS were captured and classified into three types: PMDS, other PMS studies beginning after product launch with primary data collection from medical institutions, or a post-marketing clinical trial. It is worth noting that in Japan, post-marketing clinical trials are frequently listed in the RMP to act as a mechanism by which patients who participated in pre-approval trials can continue to access the medication until it is available for their specific indication. Consequently, we excluded post-marketing clinical trials from further analysis.

### 2.3 Assessment of characteristics of PMDS and objectives

Study designs were classified as cohort, nested case-control, other, or “not decided/not described,” as explicitly mentioned in the RMP. The study with comparator group was recorded. According to the GPSP, registries were originally also included as a potential data source for PMDS, in addition to routinely collected data such as electronic medical records and administrative claims databases (Ministry of Health Labour and Welfare, 2017c). Information on the data sources employed was retrieved if specified in the RMP. Types of objectives included safety and effectiveness objectives. Effectiveness objectives were summarized. Safety objectives were considered under four categories: important identified risks, important potential risks, important missing information category, and others. Each term describing safety objectives, except for important missing information category as explicitly mentioned in the RMP, was classified under the system organ class (SOC) level of the Medical Dictionary for Regulatory Activities (MedDRA). Important missing information category is defined as information that has not been sufficiently obtained at the time of developing the RMPs. For example, this includes information necessary for evaluating the safety of the patient population that was excluded from clinical trials but which is expected to use the drug in clinical practice (Ministry of Health Labour and Welfare, 2012). Therefore, important missing information for specific patient groups was captured. We summarized the characteristics of PMDS and assessed the objectives in conjunction with the data source.

## 3 Results

### 3.1 Identification of PMDS from RMPs

**FIGURE 1:**
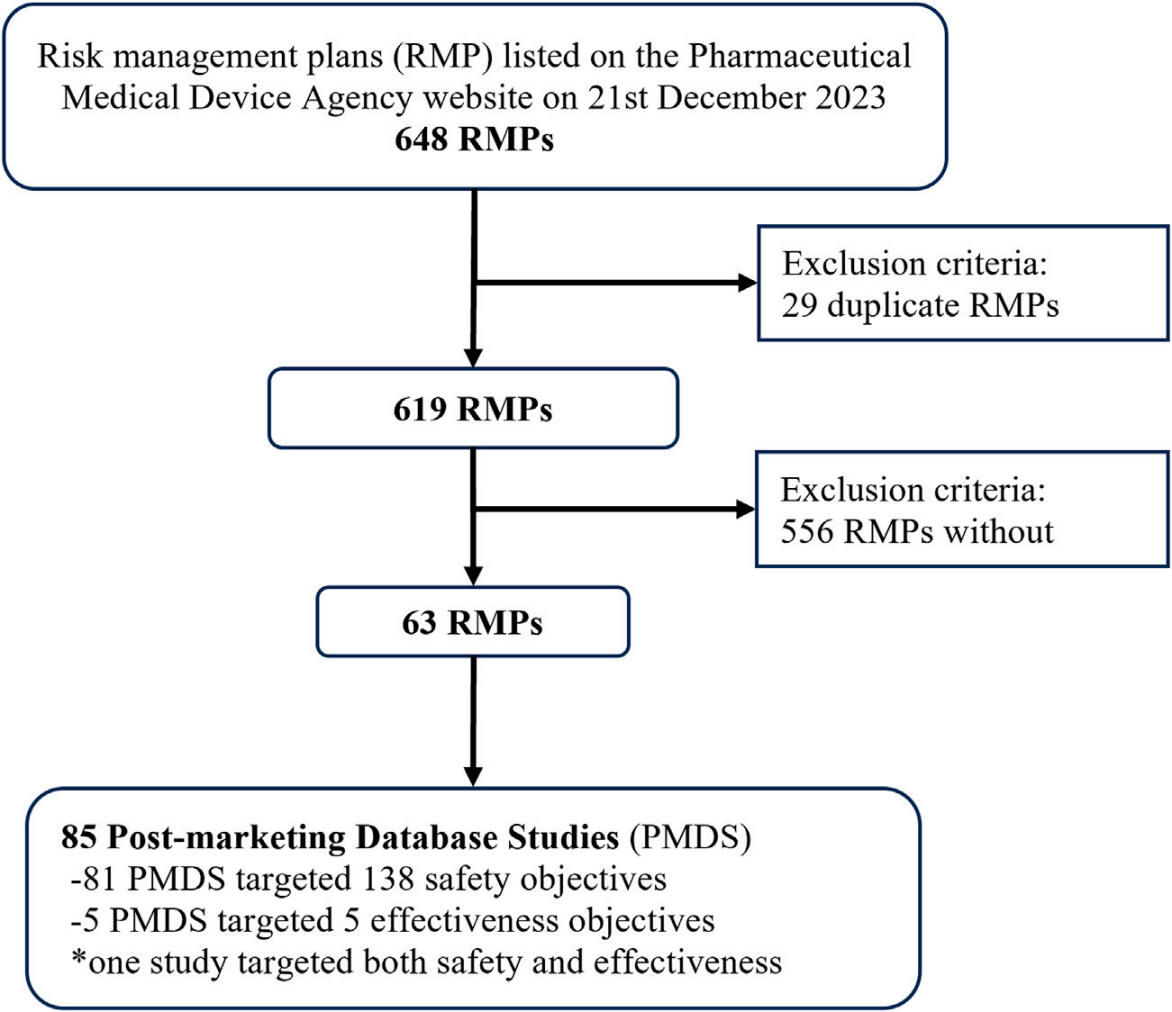
Study flow chart for identifying post-marketing database studies in risk management plans listed on the Pharmaceutical Medical and Devices Agency website.

We identified 648 RMPs on 21 December 2023, of which 29 were excluded as duplicates (each RMP covered two or more products) (Figure 1). In total, 85 PMDS were identified in 63 RMPs, with the first study approved in 2017 and the highest number of studies approved in 2019 (Supplementary Figure 1). The target objective was related to safety in 81/85 (95.3%) PMDS and to effectiveness in 5/85 (5.9%) (one PMDS targeted both).

### 3.2 Features of PMDS

Among the 85 PMDS, 57 (67.1%) related to new molecular entity, 21 (24.7%) were related to partial changes, and 7 (8.2%) to generic drugs (Table 1). Many PMDS were in RMPs reviewed by the Office of New Drugs IV (20/85, 23.5%), which is responsible for antibacterial drugs, antiviral agents, new respiratory tract drugs, anti-allergy drugs, sensory organ drugs (limited to drugs for inflammatory diseases) and anti-HIV/AIDS agents, and the Office of New Drugs I (19/85, 22.4%), responsible for gastrointestinal drugs, dermatologic drugs, hormone preparations and metabolic disease drugs. Seven (8.2%) PMDS were related to an orphan drug, and six (7.1%) to a pediatric indication.

**TABLE 1:** Characteristics of post-marketing database studies.

		Post-marketing Database Studies		Concomitant planning of other post-marketing study^a^			
				No		Yes	
		n	%	n	%	n	%
		85	100	63	74.1	22	25.9
New drug application type	New molecular entity^b^	57	67.1	38	66.7	19	33.3
	Partial change^c^	21	24.7	19	90.5	2	9.5
	Generic drug	7	8.2	6	85.7	1	14.3
Reviewing office^d^	Office of New Drugs I	19	22.4	18	94.7	1	5.3
	Office of New Drugs II	12	14.1	9	75.0	3	25.0
	Office of New Drugs III	9	10.6	6	66.7	3	33.3
	Office of New Drugs IV	20	23.5	9	45.0	11	55.0
	Office of New Drugs V	13	15.3	13	100	0	0.0
	Office of Vaccines and Blood Products	5	5.9	2	40.0	3	60.0
	Office of Cellular and Tissue-based Products	7	8.2	6	85.7	1	14.3
Re-examination period	4 years	11	12.9	10	90.9	1	9.1
	6 years	5	5.9	5	100	0	0.0
	8 years	49	57.6	31	63.3	18	36.7
	10 years	7	8.2	6	85.7	1	14.3
	Remaining period	6	7.1	5	83.3	1	16.7
	Not applicable	7	8.2	6	85.7	1	14.3
Orphan drug	Yes	7	8.2	6	85.7	1	14.3
	No	78	91.8	57	73.1	21	26.9
Paediatric indication	Yes	6	7.1	5	83.3	1	16.7
	No	79	92.9	58	73.4	21	26.6
Company type	Japanese company	33	38.8	24	72.7	9	27.3
	Foreign company	52	61.2	39	75.0	13	25.0
Drug class^e^	Alimentary tract and metabolism	8	9.4	7	87.5	1	12.5
	Blood and blood forming organs	5	5.9	4	80.0	1	20.0
	Cardiovascular system	2	2.4	1	50.0	1	50.0
	Dermatologicals	1	1.2	0	0.0	1	100
	Systemic hormonal preparations, excl. sex hormones and insulins	1	1.2	1	100	0	0.0
	Anti-infectives for systemic use	8	9.4	2	25.0	6	75.0
	Antineoplastic and immunomodulating agents	34	40.0	26	76.5	8	23.5
	Musculo-skeletal system	3	3.5	3	100	0	0.0
	Nervous system	9	10.6	6	66.7	3	33.3
	Respiratory system	5	5.9	5	100	0	0.0
	Various	3	3.5	2	66.7	1	33.3
	Not assigned	6	7.1	6	100	0	0.0

^a^ Observational studies accompanied with primary data collection adopting single cohort design and were listed on risk management plan as additional pharmacovigilance activities.

^b^ New active ingredients and/or new combination drugs.

^c^ New drug application type other than new active ingredients or new combination drugs.

^d^ Each reviewing office were responsible for following areas. Office of New Drug I: gastrointestinal drugs, dermatologic drugs, hormone preparations and metabolic disease drugs, Office of New Drug II: cardiovascular drugs, drugs to treat Parkinson’s disease, drugs to treat Alzheimer’s disease, urogenital and anal drugs, combination drugs, radiopharmaceuticals and contrast media, Office of New Drug III: central nervous system drugs, peripheral nervous system drugs, anaesthetic agents, sensory organ drugs (other than drugs for inflammatory diseases) and narcotics, Office of New Drug IV: antibacterial drugs, antiviral agents (except for anti-HIV/AIDS, agents), new respiratory tract drugs, anti-allergy drugs, sensory organ drugs (limited to drugs for inflammatory diseases) and anti-HIV/AIDS, agents, Office of New Drug V: antineoplastic drugs, Office of Vaccines and Blood Products: globulins, blood coagulation-factor products, infection prophylactic vaccines and antidotes, Office of Cellular and Tissue-based Products: regenerative medical products (cellular and tissue-based products and gene therapy products) and biosimilar products.

^e^ Classified by Anatomical Therapeutic Chemical Classification System code (ATC, code).

In addition to the 85 PMDS, 22 other PMS studies (single cohort design with primary data collection) were planned under the RMP to concomitantly investigate the same indication as a PMDS (Table 1). Most (19/22, 86.4%) of the other PMS studies were assigned to new molecular entities. One-half of these studies were in RMPs reviewed by the Office of New Drugs IV.

### 3.3 PMDS designs and data sources

**FIGURE 2:**
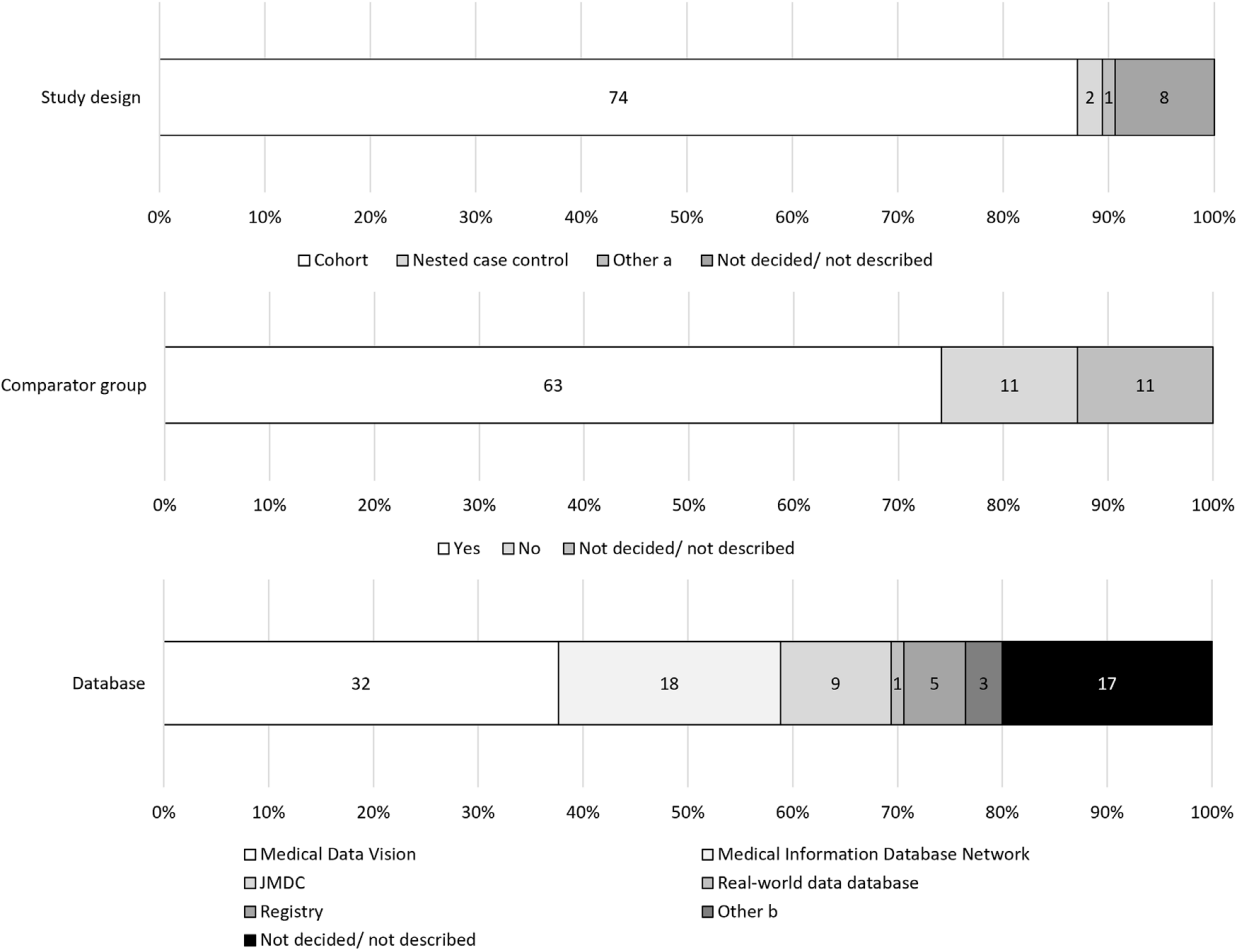
Study designs and data sources. **(a)** Matched cohort design and Sequence Symmetry design. **(b)** One study used both Medical Data Vision and Medical Information Database Network, two proposed Medical Data Vision or Medical Information Database Network as candidates.

Cohort studies were the most prevalent study design of PMDS (87.1%, 74/85), though the design of eight studies was not described (Figure 2). In total, 63 out of 85 (74.1%) PMDS described a set comparator group, and the remaining 11 PMDS had no comparator group, and in 11, the use of a comparator group was either not decided or not described. MDV was used by 32 studies (37.6%), MID-NET^®^ was used by 18 studies (21.2%), and the JMDC was used by nine studies (10.6%). The data source was not described in 17 PMDS (20.0%).

### 3.4 Target objectives and associated data sources

Among 85 PMDS, 81 (95.3%) targeted safety objectives, in which 57 (67.1%) studies targeted important identified risks and 29 (34.1%) targeted important potential risks (Table 2). In total, there were 138 safety objectives across the 81 studies, of which 86 (62.3%) were important identified risks, 34 (24.6%) were important potential risks, 16 (11.6%) were important missing information category.

**TABLE 2:** Objectives of post-marketing database studies and their classification.

	No. of post-marketing database studies		No. of objectives	
	n	%	n	%
Total number of post-marketing database studies	85	100	—	—
Safety objectives	81	95.3	138	100
Important identified risk	57	67.1	86	62.3
Important potential risk	29	34.1	34	24.6
Others^a^	2	2.4	2	1.4
Important missing information	11	12.9	16	11.6
Effectiveness objectives	5	5.9	5	100

^a^ Events which could not be classified (eg, specific adverse events during the acute phase, non-acute hospitalization events).

The most common disease targets classified according to MedDRA SOC level except for important missing information category were “Infections and infestations” (18/122, 14.8%), “Metabolism and nutrition disorders” (17/122, 13.9%), and “Cardiac disorders” and “Vascular disorders” (each 15/122, 12.3%) (Table 3). The most prevalent terms of safety objectives as explicitly described in the RMPs were serious infection (n = 9), cardiovascular event (*n* = 7), malignant tumor and hemorrhage (*n* = 6 each), and myelosuppression and hypoglycemia (*n* = 5 each) (Supplementary Table 1).

**TABLE 3:** Classification of safety and effectiveness objectives and data sources used.

	No. of objectives		Data source													
			Medical Data Vision		Medical Information Database Network		JMDC		Real-world data database		Registry		Other^a^		Not decided/ not described	
	n	%	n	%	n	%	n	%	n	%	n	%	n	%	n	%
Total number of important identified and potential risks and others^b^	122	100	47	38.5	17	13.9	8	6.6	1	0.8	23	18.9	3	2.5	23	18.9
Infections and infestations	18	14.8	8	44.4	0	0.0	1	5.6	0	0.0	3	16.7	0	0.0	6	33.3
Neoplasms benign, malignant and unspecified (incl cysts and polyps)	8	6.6	4	50.0	0	0.0	0	0.0	0	0.0	2	25.0	0	0.0	2	25.0
Blood and lymphatic system disorders	10	8.2	4	40.0	3	30.0	0	0.0	0	0.0	2	20.0	0	0.0	1	10.0
Immune system disorders	4	3.3	0	0.0	0	0.0	1	25.0	0	0.0	3	75.0	0	0.0	0	0.0
Endocrine disorders	1	0.8	0	0.0	1	100	0	0.0	0	0.0	0	0.0	0	0.0	0	0.0
Metabolism and nutrition disorders	17	13.9	5	29.4	5	29.4	3	17.6	0	0.0	0	0.0	2	11.8	2	11.8
Psychiatric disorders	1	0.8	0	0.0	0	0.0	0	0.0	1	100	0	0.0	0	0.0	0	0.0
Nervous system disorders	2	1.6	2	100	0	0.0	0	0.0	0	0.0	0	0.0	0	0.0	0	0.0
Eye disorders	1	0.8	0	0.0	0	0.0	0	0.0	0	0.0	1	100	0	0.0	0	0.0
Cardiac disorders	15	12.3	4	26.7	3	20.0	0	0.0	0	0.0	2	13.3	1	6.7	5	33.3
Vascular disorders	15	12.3	8	53.3	0	0.0	1	6.7	0	0.0	3	20.0	0	0.0	3	20.0
Respiratory, thoracic and mediastinal disorders	5	4.1	4	80.0	0	0.0	0	0.0	0	0.0	1	20.0	0	0.0	0	0.0
Hepatobiliary disorders	5	4.1	1	20.0	2	40.0	0	0.0	0	0.0	1	20.0	0	0.0	1	20.0
Skin and subcutaneous tissue disorders	1	0.8	0	0.0	0	0.0	0	0.0	0	0.0	1	100	0	0.0	0	0.0
Musculoskeletal and connective tissue disorders	1	0.8	0	0.0	0	0.0	0	0.0	0	0.0	0	0.0	0	0.0	1	100
Renal and urinary disorders	4	3.3	2	50.0	1	25.0	0	0.0	0	0.0	1	25.0	0	0.0	0	0.0
Investigations	1	0.8	0	0.0	0	0.0	0	0.0	0	0.0	0	0.0	0	0.0	1	100
Injury, poisoning and procedural complications	1	0.8	1	100	0	0.0	0	0.0	0	0.0	0	0.0	0	0.0	0	0.0
Others^c^	12	9.8	4	33.3	2	16.7	2	16.7	0	0.0	3	25.0	0	0.0	1	8.3
Total number targeting important missing information	16	100	8	50.0	2	12.5	0	0.0	0	0.0	3	18.8	0	0.0	3	18.8
Renal dysfunction	5	31.3	2	40.0	2	40.0	0	0.0	0	0.0	1	20.0	0	0.0	0	0.0
Long-term use	3	18.8	2	66.7	0	0.0	0	0.0	0	0.0	1	33.3	0	0.0	0	0.0
Safety of medication switch	3	18.8	3	100	0	0.0	0	0.0	0	0.0	0	0.0	0	0.0	0	0.0
Children	1	6.3	0	0.0	0	0.0	0	0.0	0	0.0	1	100	0	0.0	0	0.0
Pregnancy	1	6.3	0	0.0	0	0.0	0	0.0	0	0.0	0	0.0	0	0.0	1	100
Others^d^	3	18.8	1	33.3	0	0.0	0	0.0	0	0.0	0	0.0	0	0.0	2	66.7
Total number with effectiveness objectives	5	100	0	0.0	2	40.0	1	20.0	0	0.0	2	40.0	0	0.0	0	0.0
Effectiveness in clinical practice	5	100	0	0.0	2	40.0	1	20.0	0	0.0	2	40.0	0	0.0	0	0.0

^a^ One study used both Medical Data Vision and Medical Information Database Network, two proposed Medical Data Vision or Medical Information Database Network as candidates.

^b^ Classified according to system organ class (SOC) level of the Medical Dictionary for Regulatory Activities.

^c^ Diseases not categorized into the above SOCs (e.g., concomitant use with cytochrome P450 inhibitors, use in patients with liver dysfunction, specific adverse drug events in acute phase).

^d^ Patient groups not categorized into the above groups (e.g., safety of re-administration, safety in adult patients with low body weight, serious cardiovascular events).

Considering SOCs with at least four safety objectives, MDV was the most commonly used data source for all SOCs except “Immune system disorders”, “Metabolism and nutrition disorders” and “Hepatobiliary disorders” (Table 3). For “Metabolism and nutrition disorders”, where MDV was used equally with MID-NET^®^. Regarding “Hepatobiliary disorders” and “Immune system disorders”, where the use of MID-NET^®^ or registries was more common respectively. MDV was used for four out of five studies investigating serious infections, and for all studies investigating cardiovascular events and malignant tumors where the data source was specified (Supplementary Table 1).

Important missing information category mainly related to safety information in patients with renal dysfunction (5/16, 31.3%), safety information concerning long-term drug use (3/16, 18.8%) and the safety of medication switching (3/16, 18.8%) (Table 3). MDV was used for one-half of studies targeting important missing information category, including all three studies investigating the safety of medication switching, but was used equally with MID-NET^®^ for studies of patients with renal dysfunction.

None of the five effectiveness objectives were investigated using MDV but were conducted with MID-NET^®^ (*n* = 2), JMDC (*n* = 1) and Registries (n = 2) (Table 3).

## 4 Discussion

This study comprehensively describes the designs, objectives, and data sources used in PMDS approved by the PMDA in Japan since they were first introduced in 2017. Previous work by the PMDA in 2022 assessed trends in studies conducted after adoption of the 2018 GPSP revision (Kohama et al., 2023), and the Japan Pharmaceutical Manufacturers Association listed the objectives and data sources of 18 PMDS for 13 products approved in the year immediately after the GPSP revision (Takahashi et al., 2020). Our study builds on and extends these data to cover the whole period over which PMDS have been employed, and to evaluate PMDS from the perspective of study design, as well as linking target objectives to data sources.

The most common safety objectives except for important missing information category targeted by PMDS were under the SOCs “infections and infestations”, “metabolism and nutrition disorders”, “cardiac disorders” and “vascular disorders”. Regarding the terms of safety objectives as explicitly mentioned in the RMP, serious infection, malignant tumor and hemorrhage were prevalent, and these case-finding algorithms have been validated in MDV (Yamaguchi et al., 2015; Nishikawa et al., 2022). MDV was the most frequently used data source for safety objectives and was the data source in most studies investigating serious infections and malignant tumor, and where the data source was specified. Other outcome definitions, including malignant tumor, have also been validated in MID-NET^®^ (Pharmaceuticals and Medical Devices Agency, 2023).

The databases used in PMDS vary in terms of their structure, data content, and population covered. All meet the required quality standards and are commonly used for research purposes in Japan (Ministry of Health Labour and Welfare, 2018). MDV is a hospital-based database sourced from large acute care hospitals, encompassing comprehensive administrative claim data and laboratory test results for a subset of patients (Nagai et al., 2021; Laurent et al., 2022). Likewise, MID-NET^®^ integrates data from advanced large hospitals (Yamaguchi et al., 2019), which suggests it has a similar level of representativeness to MDV. Notably, MID-NET^®^ is derived from electronic medical records and includes a greater proportion of laboratory data available for analysis; however, the number of patients in MID-NET^®^ is currently smaller than that in MDV (Japanese Society for Pharmacoepidemiology, 2023; Pharmaceuticals and Medical Devices Agency, 2024). JMDC is one of the major database options derived from health insurance societies serving middle-to-large size companies (Nagai et al., 2021; Laurent et al., 2022) and data come from different sources (insurance claims) compared to MDV or MID-NET^®^. As a distinct feature, JMDC provides ledger information that enables follow-up of patients across all medical institutions where they receive care (Nagai et al., 2021). A limitation of the JMDC is that it only includes employees and their dependents up to age 75 years, reducing its applicability to older populations. All three databases may be subject to data deficiencies or errors; for example, incorrect disease coding and lack of clinical information in claims data, missing information due to incomplete medical record keeping or care received in other institutions in hospital-based databases (Suto et al., 2024). RWD may also be subject to unrecognized confounding factors. These potential limitations exist for all RWD and are not unique to databases in Japan (Baumfeld Andre et al., 2020; Liu and Panagiotakos, 2022). Database selection for individual PMDS therefore depends on matching the available sample size, data source and population characteristics with the research question–whether assessing safety or effectiveness.

The design and conduct of pharmacovigilance activities in Japan continues to advance. PMDS have been proposed for pharmacovigilance activities in Japan since the publication of the International Conference on Harmonization E2E guideline (I C H, 2004). The 2018 GPSP revision officially included PMDS as a type of PMS in Japan (Ministry of Health Labour and Welfare, 2017a; b). The PMDA reported that the proportion of PMDS had increased after the GPSP revision (Kohama et al., 2023). Nevertheless, one report indicated that only 32% of pharmaceutical companies were able to utilize databases in department responsible for PMS (Ishiguro and Uyama, 2019), possibly due to inadequate real-world evidence capabilities, and/or limited accessibility to RWD. This suggests that there is room for further expansion of PMDS into PMS.

In our study, most PMDS related to investigation of new molecular entities. This is consistent with a previous report that found all new molecular entities between April 2018 to March 2019 were approved with additional pharmacovigilance activities (Takahashi et al., 2020). However, the most common reason for conducting a PMDS in our study was to investigate an important identified risk, probably reflecting the 1980 Pharmaceutical Affairs Law in Japan (Japan Pharmaceutical Manufacturers Association, 2009), intended to describe the incidence of common adverse drug reactions in the Japanese population. In 2024, the Ministry of Health, Labour and Welfare published a notification that provides greater clarity on the procedure for formulating pharmacovigilance activities, and outlines the rationale for post-marketing activity requirements (Ministry of Health Labour and Welfare, 2024). These include investigation of causal relationships between treatment and important potential risks and determination of the important missing information category in RMPs, with investigation of important identified risks only requested if more detailed information is considered necessary.

The assessment of causal relationships of important potential risks is a relatively new expansion of pharmacovigilance activities in Japan. After the GPSP revision, one report pointed out that it was critical for pharmaceutical companies to be aware of the potential of PMDS as a method to assess causal relationship given the opportunity of including comparator groups in PMS (Ishiguro and Uyama, 2019). The Ministry of Health, Labour and Welfare and the PMDA has attempted to proceed with this transition through the publication of guidance (Ministry of Health Labour and Welfare, 2024; Pharmaceuticals and Medical Devices Agency, 2014), However, the ability of PMDS to successfully address causality objectives depends to large extent on the content and quality of existing databases, such as the availability of valid outcome definitions, the validity and comprehensiveness of available data elements, and representativeness of the database population to the target population (Ministry of Health Labour and Welfare, 2017a; b). Claim-based databases in particular, may not be appropriate to assess causal associations given they lack clinical information and information about potential confounding demographic and life-style factors such as body mass index, smoking status, and alcohol intake (Suto et al., 2024). Establishing causal associations using RWD has many technical challenges, not least the identification of appropriate control groups, and is a topic of continued interest (Lipsitch et al., 2010; D'Arcy et al., 2018). The recently published “Process guide for inferential studies using healthcare data from routine clinical practice to evaluate causal effects of drugs (PRINCIPLED)” (Desai et al., 2024) aims to address some of these challenges. Should PMDS be able to effectively address specific objectives within these limitations, the publication of the notification in 2024 may facilitate a transition from other PMS designs to PMDS in the future.

On the other hand, the variety of databases currently available for PMDS in Japan remains limited. Therefore, expanding the diversity of real-world databases will be essential for facilitating causal relationship assessments of various important potential risks. With the recent revision of the Next-Generation Medical Infrastructure Act (Cabinet Office Government of Japan, 2020), the number and diversity of medical information databases in Japan are expected to increase in the future.

There were limitations in this study. First, after completion of the re-examination period for all indications, the RMP may be deleted from the PMDA website, and as such, could not be included in our analysis. Additionally, only the latest RMP version is published on the PMDA website and information on post-marketing activities can be deleted after their completion. However, because the first PMDS were listed in 2017 and most assigned re-examination periods were at 8 years, we estimated that we were able to capture a majority of PMDS approved after 2017. Second, RMPs are a regulatory document that provide an outline of intended studies (Ministry of Health Labour and Welfare, 2012). Detailed protocols are usually finalized after consultation, review and approval by the PMDA before data analysis begins. Therefore, while little study methodology is provided in the RMP, the PMDS protocols must meet PMDA standards. However, registration and publication of the final PMDS protocol are not required and they are not usually publicly available. The PMDA’s evaluation of the detailed methodology and results of PMDS is provided in re-examination reports, which are made publicly available. However, because many of the PMDS included in this study have not yet been completed, we were unable to review the re-examination reports. Consequently, detailed methodologies for each PMDS were not available to us and we were unable to assess the validity of the choice of database or study design for the intended research question. Further research is needed to understand the appropriateness of the choice of data source and study design for investigation of individual target objectives using re-examination reports published by the PMDA after the completion of the PMDS. However, earlier public availability of protocols would be valuable for assessing the validity of PMDS and would enhance the utility of PMDS in safety monitoring.

Based on our knowledge, this is the first study to describe the characteristics of PMDS being conducted in Japan, and to specifically investigate the data sources being used to investigate specific target objectives. In Japan, PMDS were usually cohort studies with targeted safety objectives that included a comparator group. PMDS may offer advantages over single cohort prospective observational studies with primary data collection, such as very large populations, rapidity, and low cost, and can have particular utility in evaluating the occurrence of rare adverse events. The possibility to include comparator groups is an additional benefit of PMDS, because almost all PMS conducted in Japan are single cohort design. This potential advantage could make PMDS appropriate tools for assessing important potential risks, although our study indicated that many PMDS mainly targeted important identified risks. Our results could be informative for pharmaceutical companies planning to investigate specific safety or effectiveness objectives using RWD, and for planning PMDS as pharmacovigilance activities.

## Data Availability

The original contributions presented in the study are included in the article/supplementary material, further inquiries can be directed to the corresponding author.
