# Supplementary material for: Regulatory-required post-marketing database studies in Japan could be leveraged to assess important potential risks as well as identified risks
Source: Front Pharmacol. 2025 Jul 21;16:1565314. doi: 10.3389/fphar.2025.1565314 (PMC12319243; doi:10.3389/fphar.2025.1565314)
Supplement: Supplementary file 1 [file DataSheet1.pdf]

frontiers

Supplementary Material

1 Supplementary Figures and Tables

1.1 Supplementary Figure

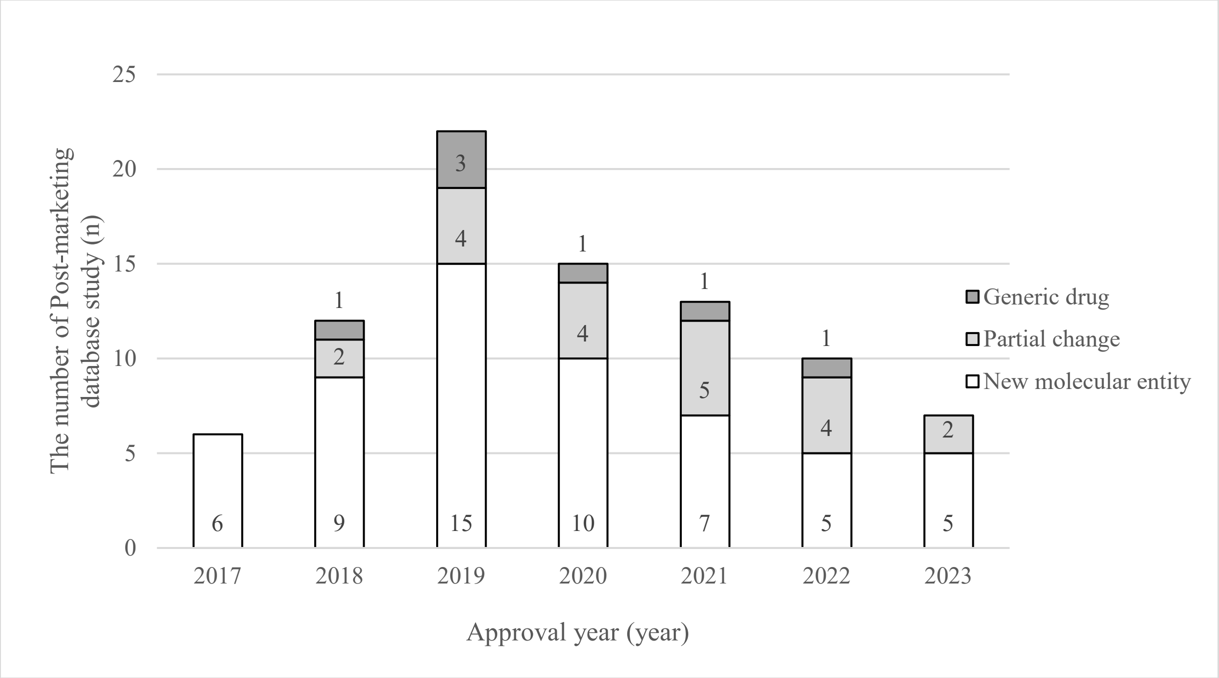

Supplementary Figure 1. The number of post-marketing database studies according to approval year

1.2 Supplementary Table

Supplementary Table 1 Important identified risks and important potential risks and others for investigation in post-marketing database studies reported in the risk management plan

Disease (system organ class level of the Medical Dictionary for Regulatory Activities)	Important identified risk/ important potential risk/ others	No. of objectives	Database name: Medical Data Vision	Medical Information Database Network	JMDC	Real-world data database	Registry	Other[a]	Not decided/ not described
Infections and infestations	Infections during chemotherapy	1	1						
	Infections	4	2				2		
	Serious infections	9	4		1				4
	Serious tuberculosis	1							1
	Pneumonia	1	1						
	Skin infection	1							1
	Meningitis aseptic	1					1		
Neoplasms benign, malignant and unspecified (incl cysts and polyps)	Malignant tumour	6	4						2
	Secondary malignant tumour	2					2		
Blood and lymphatic system disorders	Haematotoxicity during chemotherapy	1		1					
	Haematotoxicity	1	1						
	Myelosuppression	5	2	1			2		
	Bleeding tendency	1							1
	Pancytopenia, leukopenia, neutropenia, agranulocytosis, thrombocytopenia	1	1						
	Agranulocytosis, leukopenia	1		1					
Immune system disorders	Anaphylactic reaction	1					1		
	Shock, anaphylaxis	1			1				
	Hypersensitivity	2					2		
Endocrine disorders	Thyroid function decreased	1		1					
Metabolism and nutrition disorders	Ketoacidosis, effect of ketone body increased	2			2				
	Hyperkalaemia	3		2				1	
	Hyperglycaemia, diabetic ketoacidosis, diabetic coma	2		1					1
	Severe hypoglycaemia	1	1						
	Hypokalaemia	1						1	
	Hypocalcaemia	1		1					
	Hypomagnesaemia	1	1						
	Hypoglycaemia	5	2	1	1				1
	Lactic acidosis	1	1						

Disease (system organ class level of the Medical Dictionary for Regulatory Activities)	Important identified risk/ important potential risk/ others	No. of objectives	Database name						
			Medical Data Vision	Medical Information Database Network	JMDC	Real-world data database	Registry	Other[a]	Not decided/ not described
Psychiatric disorders	Hostility and aggression	1				1			
Nervous system disorders	Coma, cerebrovascular disorder, brain oedema	1	1						
	Neuropathy peripheral and optic nerve disorder	1	1						
Eye disorders	Eye disorder	1					1		
Cardiac disorders	Cardiac failure congestive	1						1	
	Ischaemic heart disease	1							1
	Serious cardiovascular event	1		1					
	Cardiovascular event	7	3						4
	Cardiac disorder	1	1						
	Heart failure	1					1		
	Heart failure, pulmonary oedema	1		1					
	Heart failure, cardiac arrhythmia	1		1					
	Cardiac arrhythmia	1					1		
Vascular disorders	Thromboembolism	1					1		
	Thrombosis, thromboembolism	1							1
	Hypertension, hypertensive crisis	2	2						
	Haemorrhage	6	3		1		1		1
	Haemorrhagic event	1					1		
	Venous thromboembolism	3	2						1
	Arterial thromboembolism	1	1						
Respiratory, thoracic and mediastinal disorders	Interstitial pneumonia, lung disorder	1	1						
	Interstitial lung disease	4	3				1		
Hepatobiliary disorders	Hepatic function disorder	2		2					
	Liver disorder	1							1
	Hepatic failure, hepatic function disorder	1					1		
	Hepatic failure, liver disorder	1	1						
Skin and subcutaneous tissue disorders	Oculomucocutaneous syndrome (Stevens-Johnson syndrome)	1					1		
Musculoskeletal and connective tissue disorders	Fracture	1							1
Renal and urinary disorders	Renal impairment	1					1		
	Function kidney decreased	1		1					
	Renal disorder	1	1						
	Proteinuria, nephrotic syndrome	1	1						
Investigations	Blood pressure decreased, syncope	1							1
Injury, poisoning and procedural complications	Infusion reaction	1	1						

Disease (system organ class level of the Medical Dictionary for Regulatory Activities)	Important identified risk/ important potential risk/ others	No. of objectives	Database name						
			Medical Data Vision	Medical Information Database Network	JMDC	Real-world data database	Registry	Other[a]	Not decided/ not described
Others	Drug interaction with CYP3A inhibitor	1					1		
	Drug interaction with CYP3A4 inhibitor	2	2						
	Use in patients with liver dysfunction	2		1			1		
	Specific adverse events during the acute phase	1			1				
	Concomitant administration of antiplatelet agent and non-steroidal anti-inflammatory drug	2	2						
	Use in patients with severe liver dysfunction (Child-Pugh C)	1							1
	Effects on transitional cell carcinoma in ureter or urinary system	1					1		
	Non-acute hospitalization events	1			1				
	Diseases suspected to be related to immunity	1		1					
Total		122	47	17	8	1	23	3	23

[a] One study used both Medical Data Vision and Medical Information Database Network, two proposed Medical Data Vision or Medical Information Database Network as candidates
